# Gluten sensitivity and epilepsy: a systematic review

**DOI:** 10.1007/s00415-018-9025-2

**Published:** 2018-08-23

**Authors:** Thomas Julian, Marios Hadjivassiliou, Panagiotis Zis

**Affiliations:** 1 Sheffield Institute for Translational Neuroscience, University of Sheffield, 385a Glossop Rd, Sheffield, S10 2HQ UK; 2 Academic Department of Neurosciences, Sheffield Teaching Hospitals NHS Trust, Sheffield, UK

**Keywords:** CEC, CD, Coeliac, Celiac, Epilepsy, Gluten

## Abstract

**Objective:**

The aim of this systematic review was to establish the prevalence of epilepsy in patients with coeliac disease (CD) or gluten sensitivity (GS) and vice versa and to characterise the phenomenology of the epileptic syndromes that these patients present with.

**Methodology:**

A systematic computer-based literature search was conducted on the PubMed database. Information regarding prevalence, demographics and epilepsy phenomenology was extracted.

**Results:**

Epilepsy is 1.8 times more prevalent in patients with CD, compared to the general population. CD is over 2 times more prevalent in patients with epilepsy compared to the general population. Further studies are necessary to assess the prevalence of GS in epilepsy. The data indicate that the prevalence of CD or GS is higher amongst particular epileptic presentations including in childhood partial epilepsy with occipital paroxysms, in adult patients with fixation off sensitivity (FOS) and in those with temporal lobe epilepsy (TLE) with hippocampal sclerosis. A particularly interesting presentation of epilepsy in the context of gluten-related disorders is a syndrome of coeliac disease, epilepsy and cerebral calcification (CEC syndrome) which is frequently described in the literature. Gluten-free diet (GFD) is effective in the management of epilepsy in 53% of cases, either reducing seizure frequency, enabling reduced doses of antiepileptic drugs or even stopping antiepileptic drugs.

**Conclusion:**

Patients with epilepsy of unknown aetiology should be investigated for serological markers of gluten sensitivity as such patients may benefit from a GFD.

## Introduction

Gluten-related disorders (GRDs) represent a spectrum of diverse clinical manifestations triggered by the ingestion of gluten. Although the gastrointestinal manifestations of gluten sensitivity are the most well studied and popularly recognised, there are a range of debilitating neurological manifestations of gluten sensitivity which are increasingly established as the cause of significant disability.

Coeliac disease (CD) is the best recognised amongst these disorders, affecting around 1% of the population [1]. CD describes patients with primarily small bowel enteropathy in genetically susceptible individuals after exposure to the protein gliadin [2]. Non-coeliac gluten sensitivity (NCGS) refers to patients with primarily gastrointestinal symptoms related to the ingestion of wheat, barley and rye who do not have enteropathy but who symptomatically benefit from a gluten-free diet (GFD) [3]. Amongst patients presenting to neurology, gastrointestinal symptoms are comparatively rare and thus this definition is unhelpful [4]. Therefore, for neurological purposes we refer to patients as being *gluten sensitive* (GS), which is defined by positive serology in the form of anti- gliadin IgG and IgA (AGA), transglutaminase (tTG) or endomysial antibodies (EMA) and the presence of a range of extra-intestinal symptoms, but without an abnormal bowel biopsy which is diagnostic of coeliac disease.

Gluten sensitivity is associated with a number of neurological conditions including ataxia [5], headaches with white matter abnormalities on MR imaging [6], peripheral neuropathy [7] and epilepsy [8]. Perhaps the first description of an association between gluten sensitivity and epilepsy was in an article published in 1956 which described a case series of ‘fits’ in children who the author described to be afflicted with behavioural and gastrointestinal disturbance linked to ingestion of gluten but without diagnostic testing [9]. Epileptic seizures presenting in the context of gluten sensitivity encompass the full spectrum of epilepsy and include both patients with and without overt brain pathology who may or may not respond to antiepileptic drugs (AED). This spectrum includes a range of interesting pathological features including a well-defined syndrome of CD, epilepsy and cerebral calcifications (CEC) [10]; hippocampal sclerosis and temporal lobe epilepsy (TLE) in the context of gluten sensitivity [11]; and those who apparently display no pathological clues to the specific cause of the epilepsy.

The aim of this study was to systematically review the current literature in order to establish the prevalence of epilepsy in patients with CD and GS, the prevalence of CD and GS in patients with epilepsy and characterise the phenomenology of the epileptic syndromes that these patients present with.

## Methodology

### Protocol

This review is not registered on a public database. It is registered on the database of dissertation projects for the MSc in Clinical Neurology at the University of Sheffield.

### Search strategy

A systematic PubMed search was performed on the 14th of December 2017. For the search, two medical subject headings (MeSH terms) were used. Term A was “coeliac” or “celiac” or “gluten”. Term B was “epilepsy” or “epileptic” or “epilepsia” or “epilepticus” or “myoclonus” or “myoclonic”. No restrictions were applied in our search strategy.

### Inclusion and exclusion criteria

Articles eligible to be included in the review were required to meet the following criteria:

1. The study subjects were diagnosed with epilepsy of unknown aetiology and gluten sensitivity.
2. The study subjects were human.
3. The study contained original data.
4. The study was available as a full text, English language article or contained utilisable information in an English language abstract.

The following were excluded:

1. Articles which remained unavailable despite attempts to contact the author and requests for access made to the British Library.
2. Studies detailing epilepsy which is secondary to another neurological insult.
3. Articles detailing gluten sensitivity which was not confirmed using positive serology as a minimum standard.
4. Myoclonus which was reference to anything other than a seizure type in the context of epilepsy.

**Fig. 1:**
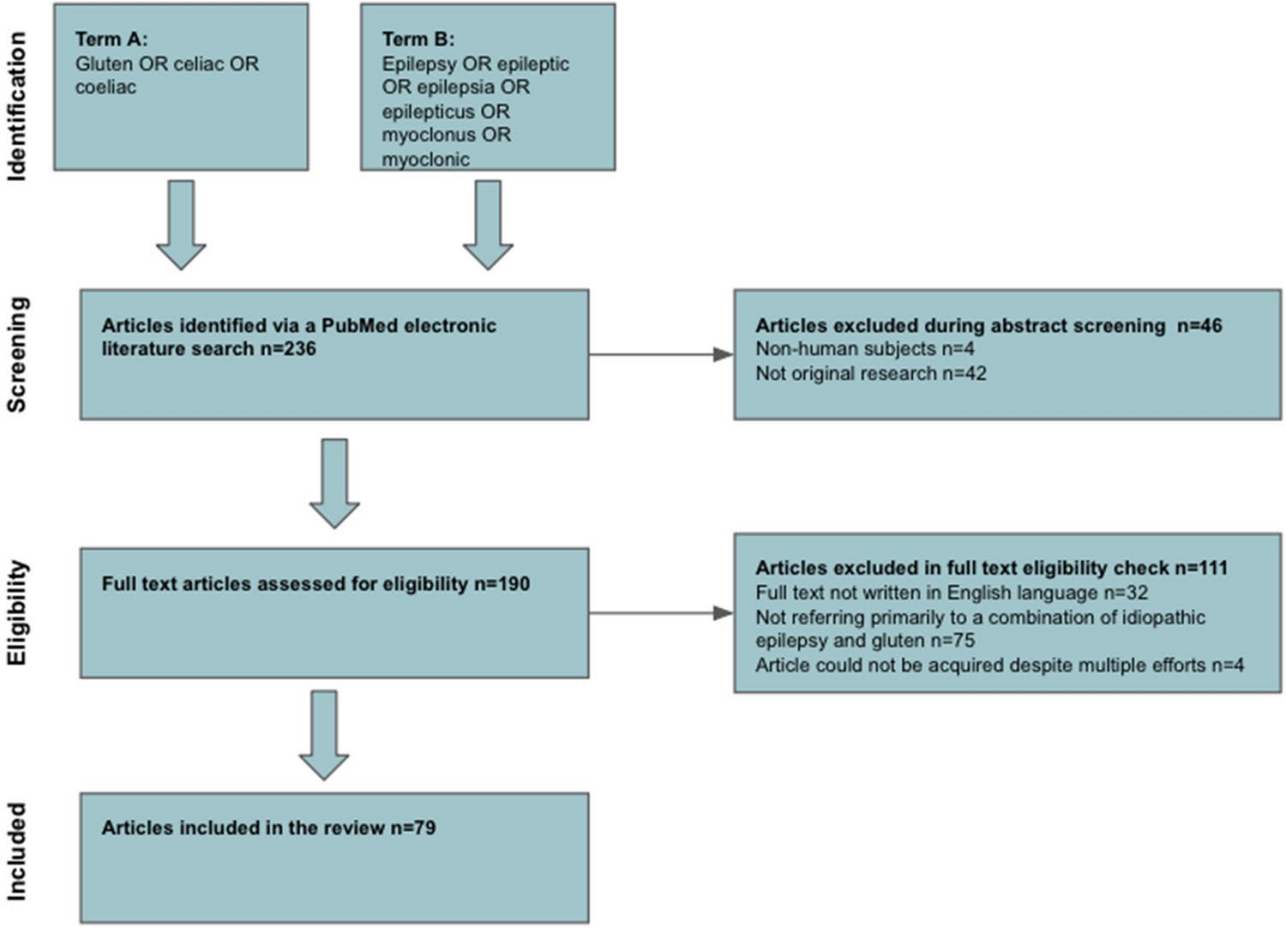
A PRISMA chart detailing the inclusion process

All studies were screened and assessed for eligibility by two authors independently. Details of the inclusion process are detailed in Fig. 1.

### Data collection process

Data were extracted from each study in a structured coding scheme using Google Sheets and included: location of study; type of study; population size, age and sex; the nature of gluten sensitivity; the classification of epilepsy; the age of onset of epilepsy and gluten sensitivity; imaging results; serological results; brain and bowel biopsy results; blood test results and epilepsy response to treatment including gluten-free diet, antiepileptic medication and surgery.

For the purposes of this review, gluten sensitivity was considered to be CD if it was categorised so by satisfying the Modified Marsh Criteria (Marsh III) [12]. Gluten sensitivity was defined as presence of one or more of the following: Positive serum AGA (IgG and/or IgA), anti-tTG or anti-EMA, without Marsh III on bowel biopsy or in the absence of biopsy. Consequently, some patients classified with gluten sensitivity who had not undergone a bowel biopsy in this review may have unidentified CD. Epilepsy of unknown aetiology in patients without a comorbid condition which may result in seizures (e.g. head injury, brain surgery, brain tumour etc) was included.

### Synthesis of results

Frequencies and descriptive characteristics extracted were calculated using Google Sheets. These were calculated as means and 95% confidence intervals (CI). This study is reported in accordance with the *Preferred Reporting Items for Systematic Reviews and Meta-Analysis* (PRISMA) guidelines.

### Assessment of bias

None of the studies included in this review are randomised control trials or interventional studies for which risk of bias tools are available. A risk of bias tool was therefore not used.

### Compliance with ethical guidelines

This article is based upon previously published studies. The article is in compliance with the journal’s ethical guidelines.

## Results

### Selected studies

The search strategy identified 236 articles. A total of 46 articles were excluded during the title and abstract screening stage. A total of 111 articles were excluded during the eligibility assessment. Thus, 79 articles published between 1970 and 2017 qualified for inclusion in this review studying a total of 39,579 individuals with either epilepsy, coeliac disease or both [8, 10, 11, 13–88]. Of these, 26 were case reports, 7 were case-control studies and the remaining 46 articles were prospective or retrospective case series (most of which were consecutively recruited cohort studies). Whilst the studies are published in a range of locations worldwide, a disproportionate number come from Italy (*n* = 24) and Turkey (*n* = 8). Figure 1 illustrates the study selection process.

### Epidemiology of gluten sensitivity and CD associated epilepsy

Thirteen studies detailed the prevalence of CD amongst patients with epilepsy of unknown aetiology [16, 21, 33, 37, 43, 45, 48, 51, 59, 63, 67, 69, 74]. The total pooled prevalence was 2.1% (95% CI 1.64–2.64%, *n* = 3389). Amongst exclusively paediatric populations the prevalence was 1.83% (95% CI 1.26–2.56%, *n* = 1804) whilst in exclusively adult populations the prevalence was 2.27% (95% CI 1.52–3.24%, *n* = 1280). Seven studies detailed prevalence of serologically confirmed gluten sensitivity amongst patients with epilepsy of unknown aetiology, and the pooled prevalence was 3.8% (95% CI 2.65–5.34%, *n* = 861) [28, 37, 41, 45, 48, 63, 68].

Fifteen studies detailed prevalence of epilepsy of unknown aetiology amongst CD sufferers [8, 13, 19, 20, 25, 38, 42, 54, 56, 57, 61, 65, 74, 86, 88]. The pooled prevalence was 1.14% (95% CI 1.03–1.26%, *n* = 33,217). Two studies detailed prevalence of epilepsy amongst serologically confirmed gluten sensitivity. The pooled prevalence was 0.93% (95% CI 0.43–1.77%, *n* = 963). Both of these figures are raised relative to a general population point prevalence of 0.64% [89].

These figures illustrate that there is an increased prevalence of CD amongst those with epilepsy and vice-versa. They also demonstrate and increased prevalence of epilepsy amongst those with GS. A large, population-based study conducted in Sweden by Ludvigsson et al. (*n* = 28,885) demonstrated a bidirectional relationship between development of CD and epilepsy which illustrates that each population is at increased risk of developing the other condition [8].

### Epilepsy classification

The classification of the epilepsy presentations was reported in 45 of the articles [8, 10, 11, 13, 15, 16, 19–22, 25–30, 32, 33, 36, 38, 41–44, 48, 49, 51, 54, 56, 57, 60–69, 74, 75, 77, 81, 86–88,, 60–69, 74, 75, 77, 81, 86–88]. Of these, 25 could be used to calculate the prevalence of focal epilepsy amongst patients with epilepsy [8, 13, 15, 16, 18–21, 28, 33, 36, 41, 43, 45, 48, 51, 54, 56, 57, 61, 63, 65, 74, 87, 88]. Amongst 351 patients, 59% presented with focal seizures. Based on this, it appears that there is a slightly greater tendency for epilepsy in the context of CD/GS to be focal [90].

### Presentation

Forty-one studies detailed the age of onset of epilepsy in those with CD/GS [11, 14–18, 22, 34–36, 39, 40, 45, 47, 52–56, 58, 60–64, 66, 71, 73, 76–80, 82, 83, 87, 70, 71, 73, 76–80, 82, 83, 87]. This gave a pooled mean age of onset of 12 (*n* = 161). Eleven studies (*n* = 52) provided information which enabled calculation of the total number of patients suffering CD and epilepsy who suffered gastrointestinal symptoms [16, 21, 33, 37, 43, 45, 48, 51, 59, 63, 64, 67, 69, 74]. This gave a pooled figure of 40% of patients having no gastrointestinal symptoms and who, therefore, only presented with neurological symptoms resulting in the diagnosis of CD. This is comparable to other neurological manifestations of gluten sensitivity in which gastrointestinal symptoms are rarely present [4]. Nevalainen et al. investigated the risk of death in patients suffering epilepsy who also had serum markers indicative of CD/gluten sensitivity and found that there was no increased risk of death in this patient cohort relative to those who have epilepsy but no gluten sensitivity [23].

### Management

Thirty-two papers discuss the therapeutic impact of gluten-free diet [10, 13, 15–18, 22, 31, 34, 36, 39, 45–47, 50, 53, 55, 59–61, 66, 67, 71, 73, 77, 80, 81, 83–85]. For the purpose of this review, a positive response was defined as either decreased frequency of seizures with GFD, cessation of seizures with GFD, successful reduction of AED with initiation of GFD or cessation of AED following the introduction of GFD. Amongst studies which reported response to GFD in a consecutively recruited series, 53% of patients were responsive to a GFD and the remaining 47% deemed unresponsive (*n* = 34) [13, 15, 16, 36, 49, 61, 67]. The response to GFD could reflect resolution/reduction of a neurological insult caused by gluten ingestion or be the result of improved absorption of AED due to resolution of gastrointestinal disturbance [69]. Data regarding response to AED alone is only available for the CEC syndrome subset. These data indicate that GFD can be an effective management of seizures in patients with gluten sensitivity.

### CEC syndrome

#### Presentation

Of the 79 studies included in this review, 30 detailed cases of CEC syndrome making it the best classified presentation of epilepsy in the context of CD and gluten sensitivity [10, 14, 17, 32, 34, 36, 44, 50, 52, 55, 60, 62, 64, 66, 70–85]. Sixteen of these papers are case reports and 14 are retrospective or prospective case series. These patients generally have focal, medically refractory epilepsy and show parieto-occipitally brain calcifications on CT or MRI. In 82% of cases, cerebral calcifications were located posteriorly (*n* = 131). In the remainder, calcifications were either frontal, temporal or in a very small minority in sub-cortical areas. The classification of epilepsy was reported in 28 articles, 78% of seizures in CEC syndrome reported in the available literature are focal in nature (*n* = 121) [14, 17, 32, 34, 36, 50, 52, 55, 60, 62, 64, 66, 70–85]. Fourteen studies detailed further localisation of the focal epilepsy and 71% of this was occipital in nature (*n* = 85) [14, 17, 36, 50, 52, 55, 60, 64, 71, 74, 76, 81, 83, 85]. Our search identified just one study that investigated a cohort of GFD treated, mostly paediatric CD patients for brain lesions independent of neurological signs [65]. The authors found no calcifications on CT, but MRI showed bilateral or unilateral T2-hyperintensive white-matter lesions in 20% of patients (*n* = 75). These lesions were independent of specific neurological presentations. This study does not support an increased prevalence of cerebral calcifications in CD patients but does demonstrate frequent brain imaging abnormalities in CD. The failure to identify calcifications is not surprising considering the rarity of CEC syndrome.

#### Pathology

Several studies show that despite improvements in seizure control with GFD there is no reduction in calcification size [10, 71, 73, 80]. This has led some researchers to suggest that the calcifications may represent an epiphenomenon rather than being causative. One speculated that the cause of the development of calcifications is folate deficiency. Primarily, this link has been made due to the co-occurrence of folate deficiency and cerebral calcifications in other situations, for instance during methotrexate therapy or in primary cerebral folate deficiency [34]. Calvani et al., have previously demonstrated decreased folic acid transport across the blood brain barrier as well as decreased intestinal absorption of folic acid in case report and speculate this to be part of the pathological process [66]. Whilst there are indeed numerous examples of co-occurring folate deficiency and CEC syndrome [10, 34, 44, 52, 71, 73, 74, 76–82], it is not a universal finding and there are numerous examples of patients with CEC syndrome and folate values within the normal range [44, 64, 79, 81]. Therefore, the relationship between folate and calcifications is inconsistent.

A case report by Johnson et al., identified high levels of IgA directed by transglutaminase isoenzyme 6 (TG6) in the patient’s serum [34]. Raised levels of TG6 have previously been shown to be elevated in other neurological manifestations of gluten sensitivity and represents a potential marker of neurological involvement [91]. This study also used indirect immunofluorescence on monkey brain which identified cerebral and cerebellar binding of IgA which demonstrates potential autoimmunity to the parenchyma of the brain. Brain biopsy was conducted in CEC syndrome patients in two case reports. One case reported a biopsy following right anterior temporal lobectomy with pathology confirming hippocampal sclerosis with severe cell loss and gliosis in CA1/CA3/CA4 [17]. The second case report by Bye et al., demonstrated cortical vascular abnormality with patchy pial angiomatosis, fibrosed veins and large, jagged microcalcification [76].

#### Management

The more descriptive nature of the CEC syndrome studies enables insight into the patient’s response to treatment. Response to AED alone appears to be poor amongst patients with CEC syndrome, with 73% unresponsive to treatment (*n* = 74) [10, 32, 36, 44, 60, 64, 74, 75, 77, 81]. Response to GFD appears to be more effective, with 53% of patients managed with GFD demonstrating a good response (*n* = 47) [10, 36, 60, 77, 81]. Interestingly, three prospective cohort studies appear to have demonstrated an inverse relationship between effectiveness of GFD and duration of epilepsy prior to GFD, perhaps due to increasing neurological damage [36, 60, 81].

There are four reported cases of surgical resection of cerebral calcifications with the aim to resolve intractable seizures [17, 36, 52, 77]. Of these, one was resistant to surgery, two were responsive to surgery and one responded to a combination of surgical resection and GFD. Whilst two patients did respond to surgery, one continued to suffer seizures but of lesser severity, and one was commenced gluten-free diet immediately following surgery. Consequently, it is feasible that the improvement is owed to GFD rather than to surgical resection. Based on the very limited data available, surgical resection of calcifications alone does not resolve epilepsy in CEC syndrome. These findings must be interpreted with caution as the data include studies using selective populations (non-consecutive case series and case reports).

### Other specific epilepsy syndromes in GS and CD

A small number of papers have investigated specific epilepsy syndromes in relation to GS and CD. These studies are limited by their small population sizes but demonstrate a possible interesting link between GS/CD and some specific syndromes.

#### Childhood partial epilepsy with occipital paroxysms

Three articles investigated the prevalence of CD amongst those with childhood partial epilepsy with occipital paroxysms [21, 26, 64]. According to these, the pooled prevalence of CD amongst such individuals is 10.41% (95% CI 5.11–18.32%, *n* = 96). This is considerably higher than the prevalence CD amongst all epilepsy of unknown aetiology.

#### Children with occipital lobe epilepsy

Dai et al. conducted a case-control study with a cohort of children with both generalised and focal epilepsy identified to be occipital in origin on EEG [27]. This study discovered 2.22% (*n* = 90) of the patient cohort also had CD which was significantly higher than the healthy control group. The two patients with CD were also monotherapy resistant.

#### Adult patients with fixation off sensitivity (FOS)

Fattouch et al. investigated adult epileptic patients with FOS [32]. These patients all presented with simple and complex focal seizures, which were posterior in onset according to EEG. This study showed a prevalence of CD of 20% (*n* = 15) amongst this patient group.

#### Progressive myoclonic epilepsy (PME)

Franceschetti et al., investigated comorbid conditions amongst patients with PME of undetermined cause by collecting existing medical record data [29]. This study identified that 0.98% (*n* = 204) of the patient cohort suffered comorbid CD. This data is limited because as previously established most patients are not symptomatic for CD, and so it is likely checking medical records alone is not sufficient to rule out CD and GS as the condition will likely be undiagnosed.

#### Temporal lobe epilepsy (TLE) with hippocampal sclerosis

Peltola et al. studied the relationship between temporal lobe epilepsy (TLE) with hippocampal sclerosis, and gluten sensitivity [11]. Autoimmunity has previously been implicated in the pathophysiology of hippocampal sclerosis and thus this study investigated GS antibodies in a prospective study of patients with refractory TLE with or without hippocampal sclerosis as well as in those with extra-temporal epilepsy. This study identified a significantly raised prevalence of gluten sensitivity seropositivity in those with TLE and hippocampal sclerosis with a figure of 43.75% (*n* = 16). These patients were then tested for CD, with three patients displaying histology for CD (18.75%) and four showing early changes associated with CD. This is the only study linking gluten sensitivity or CD to hippocampal sclerosis and TLE. This study is limited by its small population. As previously mentioned, hippocampal sclerosis was also present in the biopsy of a patient suffering CEC syndrome in a separate study [17].

#### Summary

In tandem with studies regarding CEC syndrome, these data appear to support a hypothesis of a selective vulnerability of specific brain regions to damage in the context of gluten sensitivity or coeliac disease.

### Assessment of bias

None of the studies included in this review are randomised control trials or interventional studies for which risk of bias tools are available. All articles were reviewed by two authors independently and their judgements regarding inclusion matched in all cases. Case reports are low level evidence with a high risk of bias. The use of this data is summarised in the “Limitations” section of this review.

### Conclusions

This systematic review has identified the following key points:

1. There is an increased prevalence of CD amongst patients with epilepsy and an increased prevalence of epilepsy amongst those with CD or gluten sensitivity.
2. Patients with CD presenting with neurological symptoms often suffer no gastrointestinal symptoms.
3. There appears to be a stronger link between some epileptic presentations and GS or CD than others. Future studies should not treat epilepsy as though it is homogenous when investigating its relationship with GS or CD.
4. Gluten-free diet is an effective management of epilepsy in those with epilepsy due to GS/CD.
5. CEC syndrome is the best characterised epileptic presentation linked to CD.
6. Patients with epilepsy of unknown aetiology should have their serum screened for AGA, anti tTG and EMA. This is especially important in patients suffering with AED resistant occipital lobe seizures. It is likely that there are many patients who are being treated with AED polytherapy who can be managed with a GFD alone or with GFD and reduced AED.
7. There is a need to study the prevalence of TG6 antibodies in patients with epilepsy to identify whether anti-TG6 could be used to identify individuals at risk of epilepsy due to their gluten sensitivity.

The results of this review are highly relevant to Dieticians and Gastroenterologists as well as Neurologists. It is important that epilepsy is more broadly recognised within the spectrum of gluten-related disorders as these patients can be managed effectively if identified. However, clinicians must approach these cases with caution so as to not incorrectly diagnose epilepsy in those with gluten intolerance who have epilepsy mimics such as syncope, psychogenic non-epileptic seizures or migraine, amongst others. Clinicians must also recognise the limitations to the specificity of the GS/CD serum markers and take into account the full clinical picture when proceeding with diagnosis.

## Limitations

- There is a significant deal of low quality evidence utilised in this review due to the large number of case reports. Studies which use selected patient groups were utilised to evaluate: Response to treatment, descriptive peculiarities where it is explicitly stated that a case report was used and for results regarding CEC syndrome. All other results were calculated using studies with consecutively recruited patient cohorts or entire populations only.
- Not all studies biopsied their GS patients. The patients may therefore have suffered CD. This may cause underestimation of the link between CD and epilepsy.
- Some studies reviewed medical records rather than prospectively investigating for CD/GS. Often patients do not suffer classical symptoms of GS/CD and therefore will not be diagnosed. Therefore, the prevalence of CD/GS amongst epilepsy may be an underestimate.
- This study addressed the relationship between CD/GS and epilepsy of unknown aetiology. There were no restrictions placed upon date of publication of articles included in this review and as such some studies are dated as far back as 1970. As the understanding of the causes of epilepsy has developed it is possible that some patients included within the studies forming this review would now be recognised to have a clear aetiology and therefore will be incorrectly classified.
- Four articles were excluded because they could not be retrieved.
- Articles for inclusion in this review were retrieved via a search in a single electronic database.
